# Emphysematous Pyelonephritis Following Ureterovesical Reimplantation for Congenital Obstructive Megaureter. Pediatric Case Report and Review of the Literature

**DOI:** 10.3389/fped.2019.00002

**Published:** 2019-01-24

**Authors:** Vincenza Girgenti, Gloria Pelizzo, Salvatore Amoroso, Gregorio Rosone, Marco Di Mitri, Mario Milazzo, Salvatore Giordano, Rosaria Genuardi, Valeria Calcaterra

**Affiliations:** ^1^Pediatric Surgery Department, Children's Hospital G. Di Cristina, ARNAS Civico-Di Cristina-Benfratelli, Palermo, Italy; ^2^Infectious Diseases Unit, ARNAS Civico-Di Cristina-Benfratelli, Palermo, Italy; ^3^Pediatric Intensive Care Unit and Trauma Center, Children's Hospital G. Di Cristina, ARNAS Civico-Di Cristina-Benfratelli, Palermo, Italy; ^4^Pediatrics and Adolescentology Unit, Department of Internal Medicine, University of Pavia and Fondazione IRCCS Policlinico San Matteo, Pavia, Italy

**Keywords:** emphysematous pyelonephritis, children, infant, pediatric surgery, congenital obstructive megaureter, ureterovesical reimplantation

## Abstract

**Introduction:** Emphysematous pyelonephritis (EPN) is a rare, life-threatening necrotizing infection of the kidney. To date, very few cases of EPN have been described in the pediatric age. The first case of EPN in a toddler occurring after ureterovesical reimplantation for congenital obstructive megaureter is reported with a literature review.

**Case Report:** A 23-month-old male, with a prenatal diagnosis of obstructive megaureter and incomplete duplication of the left urinary tract, was admitted to our Unit where he underwent surgery to treat increased dilatation of the renal pelvis and appearance of an obstructive curve. The latter was revealed at renal scintigraphy, the exam highlighted the radiographic aspect of the cortical renal parenchymal sufferance. At admission preoperative exams were normal, and no recurrent urinary tract infections were documented. Surgical removal of the left stenotic ureteral common tract of the incomplete duplex collecting system was accomplished; ureterovesical reimplantation was performed without ureteral recalibration. No intraoperative complications were recorded. In the immediate postoperative period, urosepsis and the patient's lethargic condition led to life-threatening conditions requiring urgent admission to the intensive care unit. Biochemical analysis showed leukocytosis, anemia, increased C-reactive protein, prolonged prothrombin time, pancytopenia, hyponatremia. Abdominal sonographic evaluation revealed the presence of gas in the left kidney. Unilateral EPN (Class 2) was confirmed by CT- scan. *Escherichia coli* was cultured from peripheral blood and antimicrobial therapy was started. No additional interventions were required. The child was discharged 14 days postoperatively with normal renal function.

**Conclusion:** EPN is a serious condition that can occur after surgical treatment for urinary tract obstruction. Early detection of air in the kidney should be considered a sign of complicated urinary tract infection. Immediate aggressive resuscitation and antimicrobial therapy are effective and curative with a positive outcome.

## Introduction

Emphysematous pyelonephritis (EPN) is an acute severe necrotizing infection of the renal parenchyma and its surrounding tissues, which results in the presence of gas in the renal parenchyma, collecting system, or perinephric tissue (1, 2).

The pathogenesis of EPN remains unclear. Some clinical conditions predispose to this entity, such as diabetes mellitus, urinary tract obstruction, and immune-incompetence (2, 3). The EPN clinical course can be severe and life-threatening if not recognized and treated promptly. Computed tomography allows for a prompt diagnosis by revealing gas accumulation in the kidney. Surgical measures and antibiotic therapy are the principal therapeutic methods (4, 5).

## Background

To date, very few cases of EPN have been described in the pediatric age (6–11). Here, we report the first case of EPN in infancy occurring after urological surgery for obstructive megaureter. A review of the literature regarding pediatric EPN cases is also reported.

## Case Report

A 23-month-old male, with left obstructive megaureter and an incomplete duplex collecting system was admitted to our Pediatric Surgery Unit for surgical treatment. The patient was born full-term by cesarean section at the 40th week of pregnancy (birth weight 3,850 g). Prenatally, at 31 weeks' gestation, a duplicated collecting system and ureter dilatation was suspected. In the first months of life, the diagnosis was made with a diethylenetriaminepentacetic acid (DPTA) renogram and confirmed by computed tomography (CT). A cystourethrogram showed no evidence of vesico-ureteric reflux.

Indications for surgery were based on a 12 month follow-up, where the following were observed: increased dilatation of the renal pelvis (34 vs. 13 mm), appearance of an obstructive curve upon DPTA diuretic renal scintigraphy, without upper kidney resolution following the administration of furosemide and the thin radiographic aspect of the cortical renal parenchyma.

Prior to admission, recurrent urinary tract infections were not documented. Preoperative (2 days pre-surgery) blood examinations and urine dipstick were normal. At admission, the patient was in good condition.

Correction of the megaureter included an open surgical approach. Through the transvescical mobilization of the megaureter, the distal narrowed common ureter with an incomplete duplex system (3–4 cm in length) was excised in order to free both ureters. No difficulties were encountered in mobilizing the ureters and extravescical ureteral exploration was not considered necessary. Both ureters measured 1 cm in diameter and ureteral plication was not performed. The ureters were reimplanted in a generous vescical submucosa tunnel, about 4 cm in length, using the Cohen Technique. The new ureteral orifices appeared large in size and ureteral stents were not necessary. A balanced electrolyte solution (5 ml/kg/h) for fluid therapy during anesthesia was infused. At the end of the operation only an urinary catheter was left in place. Resection of the ureterovesical junction of the obstructive megaureter was performed followed by common sheath vesicoreteral reimplantation (operative time: 2 h and 40 min). There were no intraoperative complications. After the procedure, no variations in diuresis were noted (3–4 ml/kg/hour).

A few hours postoperatively, the patient developed a fever (39°C), lethargy, abdominal pain, nausea and tachycardia (heart rate 170/min) with a blood pressure of 110/70 mmHg. A complete blood count showed leukocytosis (17.29 × 10^9^/L) with an 80% neutrophil predominance, anemia (hemoglobin 7.6 g/dl) and a normal platelet count (134 × 10^9^/L). C-reactive protein was elevated (19.52 mg/dl) and prothrombin time was prolonged (21.7 s). Acute kidney injury (serum creatinine 0.58 mg/dl) and hyponatremia (Na 130 mmol/L) were also detected. Fasting blood sugar was 56 mg/dl. The urine dipstick (measuring urine from a urinary Foley catheter) revealed leucocyturia, but his urine culture resulted negative. *Escherichia coli* was cultured from peripheral blood.

**Figure 1:**
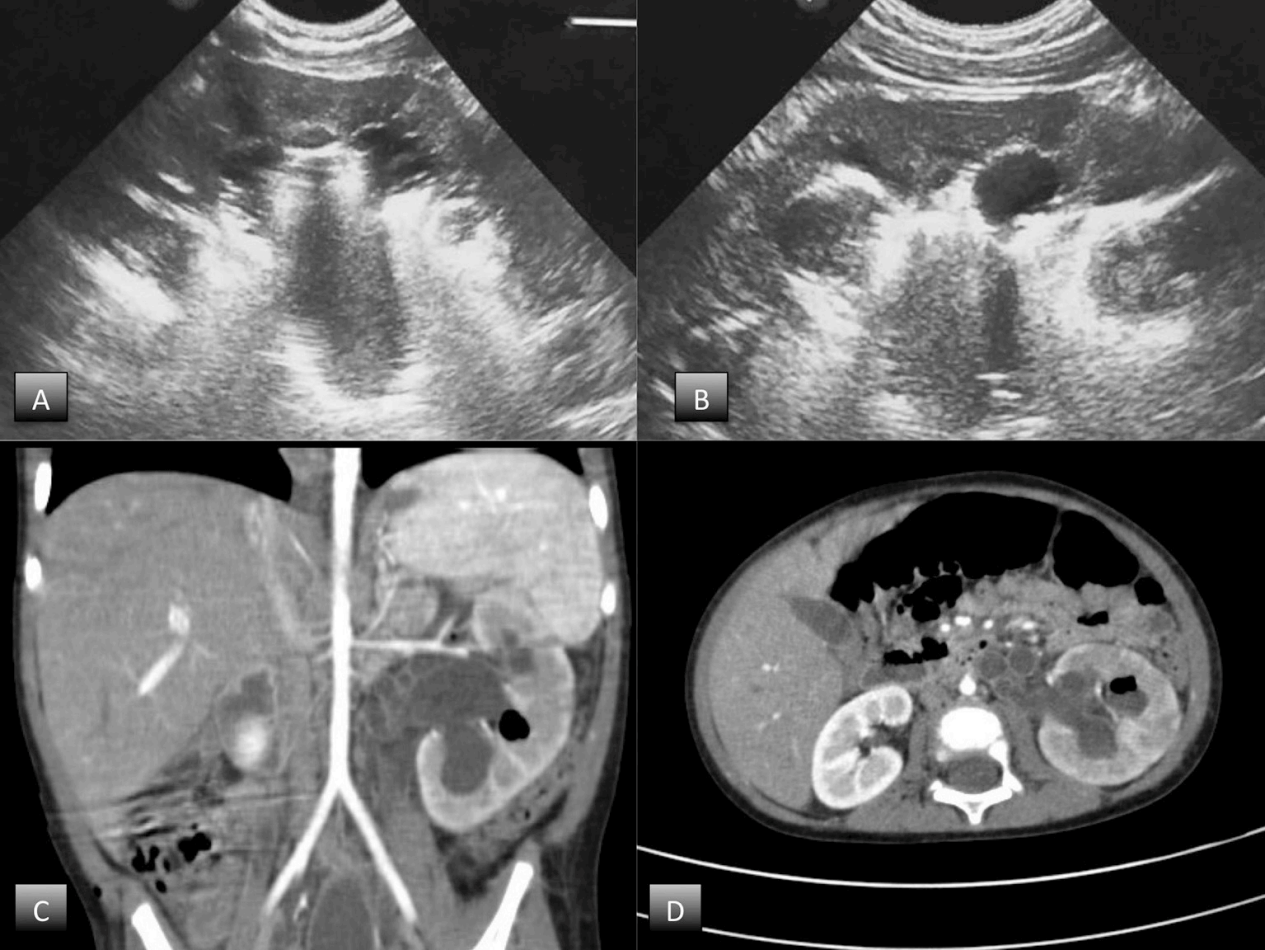
Radiological findings. **(A,B)** abdominal ultrasound; **(C,D)** abdominal CT scan with contrast.

Abdominal ultrasound demonstrated an enlarged left kidney with grade 3 hydroureteronephrosis and highly reflective echoes consistent with the presence of gas, suggestive of EPN (Figures 1A,B). An abdominal CT scan with contrast confirmed the diagnosis of unilateral EPN and showed an enlarged, hydronephrotic left kidney with discrete amounts of gas in the pelvis, lower calyceal group, and renal parenchyma (Class 2) EPN according to the Huang and Tseng classification (12) (Figures 1C,D).

The patient received fluid resuscitation in the intensive care unit. Intravenous antibiotic therapy was administered: empiric antimicrobial therapy with gentamicin (4 mg/kg/die) and ceftriaxone (50 mg/kg/die) was initially started and subsequently based on the antibiogram treatment was changed to meropenem (40 mg/kg every 8 h). Blood and plasma transfusions were also required.

Considering the complex malformation including the duplex collecting system, conservative treatment was preferred. The patient's clinical status improved significantly with medical treatment. On the fifth postoperative day he was readmitted to our surgical unit. Seven days postoperatively, a follow-up sonogram showed resolution of gas in the kidney.

He was discharged 14 days postoperatively, with a normal serum creatinine, decreased inflammatory index values and clear urine. He is being followed up at our unit and the pediatric nephrology unit.

## Discussion

EPN is a rare and life threatening suppurative infection of the renal parenchymal and perirenal tissues, characterized by spontaneous gas production (13). Since the first case reported by Kelly and MacCallum (12) in 1898, more than 600 cases of EPN have been reported in the adult age (14–17). However, the first reported case in the literature regarding a pediatric patient was in 1985 in a 10-year-old female patient (6); and to date, as reported in Table 1, only seven other cases have been reported in children (6–11). We describe the first case in which unilateral EPN was diagnosed following an urologic surgical procedure.

**Table 1:** Literature review regarding emphysematous pyelonephritis in the pediatric age.

	**Sex**	**Age**	**Diabetes**	**Other risk factors**	**Radiological findings according to Huang and Tseng (5)**	**Pathogen isolated**	**Treatment**	**Outcome**
Pode et al. (6)	F	10 years	No	Neurogenic bladder	Class 3A	*P. mirabilis, E. coli, Streptocossus fecalis*	Antibiotics +percutaneous drainage	Good
Fernandes et al. (7)	M	6 years	No	Pelviureteric junction obstruction	Class 1	Not done	Not done	Good
Al-Makadma (8)	M	12 months	No	Neurogenic bladder	Class 1	*E. coli*	Antibiotics	Good
Siddique et al. (9)	F	3 months	No	Obstruction due to ectopic right ureter	Not done	*Enterobacter cloacae*	Antibiotics	Good
Ambaram et al. (11)	M	9 years	No	Not done	Class 4	*E. coli*	Percutaneous drainage + peritoneal dialysis	Death
	F	34 months	No	Acquired immunodeficiency	Class 3	*E. coli*	Antibiotics+ Percutaneous drainage+ Laparoscopic nephrectomy	Good
Gross (10)	F	4 years	No	Renal stone	Class 1	*E. coli*	Antibiotics	Good
Our case	M	23 months	No	Nephrourological congenital malformation Surgery	Class 2	*E. coli*	Antibiotics	Good

The pathogenesis of EPN is still unknown. Several key factors have been proposed including the presence of gas-forming bacteria, high levels of glucose in the tissues, impaired tissue perfusion, reduced host immunity and urinary tract obstruction (5, 18). About 90% of adult cases have been reported in diabetics, especially in females (2). Therefore, it is thought that hyperglycaemia which results in renal vasculopathy, renal neuropathy, and leukocyte dysfunction (5, 18) is a major predisposing factor for EPN; obstructive uropathy has been another contributing factor in other cases. In the pediatric population (6–11), no children with diabetes have been described. Obstructive uropathy, stones and decreased host immunity have been the major risk factors for pediatric EPN. As reported in the literature, EPN may occur in patients with no previous recurrent infectious disease as in the case of severe obstructive congenital uropathy (7, 9). No association has been reported between preoperative urine cultures and outcomes after vesicoureteral reimplantation, suggesting that asymptomatic patients do not require preoperative surveillance (19). Our case confirms this opinion supporting that also asymptomatic patients with urinary tract obstruction are at risk of complicated postoperative urinary tract infections (20). However, the causative role of renal perfusion impairment during surgery should not be fully excluded.

*Escherichia coli* is the most common pathogenic organism found in about 70% of cases (2, 20). Other organisms such as *Proteus mirabilis, Klebsiella pneumoniae, Group D Steptococcus*, and coagulase negative *Staphylococcus* have also been found. In rare cases, anaerobic microorganisms including *Clostridium septicum, Candida albicans, Cryptococcus neoformans* and *Pneumocystis jiroveci* have also been indicated as the causative EPN pathogen (2, 5, 20–22).

Patients with EPN frequently complain of symptoms typical of pyelonephritis. In more severe conditions, as in our infant, acute renal failure and/or septic shock may occur (15). Laboratory examination usually shows increased white blood cells and thrombocytopenia, while blood glucose may be high due to coexisting diabetes mellitus (15).

Imaging is necessary for the EPN diagnosis (5, 20). A radiograph of the kidney may show trapped gas or a diffusely punctuated kidney with gas along the renal pyramids. Ultrasound may show an enlarged kidney with high amplitude echoes in the region of the kidneys with distal shadows of low magnitude echoes and reverberations “dirty shadows,” in contrast with “clean shadows” seen when renal stones are present (23). CT is the recommended choice of investigational instrument as it better estimates the amount of gas, the destruction of the renal parenchyma, the presence of collected fluid and fluid-gas levels as well as the underlying cause of the urinary tract obstruction (5, 15, 20).

There are three classifications of EPN based on radiological findings (1, 5, 24). Michaeli et al. (1) classify EPN based on the findings of plain abdominal film and intravenous pyelogram of the kidney, ureter and bladder. Based on CT findings, Wan et al. (25) categorized EPN into two types. Huang and Tseng (5) have published a more detailed classification, which is also based on CT findings, but includes more sub-categories than that of Wan et al. They classify EPN as follows: Class 1, gas is confined in the pyelocalyceal system only; Class 2, gas is found in the renal parenchyma; Class 3A, gas extends into the perinephric space; Class 3B gas extends into the pararenal space; Class 4 EPN affects a solitary kidney or the infection is bilateral. The detailed classification of Huang and Tseng (5) correlates the class of EPN and its management.

Although it is a life-threatening illness with a mortality rate of up to 50% there is no consensus on the best management of patients (25). The main focus of treatment is urgent percutaneous drainage (PCD), or medical management with or without stenting of the urinary tract (26). Medical management of EPN is multidimensional and requires vigorous resuscitation, fluid and electrolyte replacement, correction of acid-base imbalances, and antibiotic regimen. Some patients may require inotropes or even renal replacement therapy (17, 19). Regarding surgical treatment, aggressive therapy such as an urgent nephrectomy has historically been considered the treatment of choice. Current strategies suggest a nephron sparing approach involving PCD initially and an elective nephrectomy at a later stage if required (5, 15, 24, 27–29).

Huang and Tseng (5) propose surgical management based on their radiologic classification. For class 1 and 2 they suggest antibiotic terapy in combination with PCD as well as relief of urinary tract obstruction when present, with a 100% cure rate. The same approach is proposed for classes 3 and 4 when the patient has <2 risk factors, which include thrombocytopenia, acute renal function impairment, disturbance of consciousness or shock, with 85% success rates. On the contrary, when a patient has more than two risk factors, nephrectomy has the best management outcome. In the pediatric clinical literature, nephrectomy has been described in only one patient, antibiotic therapy in combination with PCD in two cases and medical treatment in the other cases. In our patient, considering the complex malformation, we preferred conservative management and obtained good results.

## Concluding Remarks

In conclusion, EPN is a serious condition that may occur after surgical treatment of congenital urinary tract obstruction. The abdominal CT plays a crucial role in confirming the diagnosis and studying the extent of the lesion and thus contributes to therapeutic decisions. Early and aggressive resuscitation and antimicrobial therapy may be effective and curative.

## Ethics Statement

Written informed consent was obtained from the patient's parents for publication of this case report and accompanying images.

## Author Contributions

All authors listed have made a substantial, direct and intellectual contribution to the work, and approved it for publication.

### Conflict of Interest Statement

The authors declare that the research was conducted in the absence of any commercial or financial relationships that could be construed as a potential conflict of interest.
